# Pioneers of modern brain research—Cécile and Oskar Vogt and the Nobel Prize

**DOI:** 10.3389/fnana.2025.1679993

**Published:** 2025-09-22

**Authors:** Nils Hansson, Heiner Fangerau, Fabio De Sio, Ursula Grell, Katrin Amunts

**Affiliations:** ^1^Department of the History, Philosophy, and Ethics of Medicine, Medical Faculty, Heinrich-Heine University Düsseldorf, Düsseldorf, Germany; ^2^Cécile and Oskar Vogt Institute for Brain Research, Medical Faculty, University Hospital Düsseldorf, Düsseldorf, Germany; ^3^INM-1, Research Centre Jülich, Institute of Neuroscience and Medicine, Jülich, Germany

**Keywords:** myeloarchitecture, brain mapping, research concepts, history of neuroscience, brain research

## Abstract

This article explores the complex and ultimately unsuccessful Nobel Prize trajectories of Oskar (1870–1959) and Cécile Vogt (1875–1962), as well as their ongoing scientific legacy. Their legacy sheds light on the background to the decision from different perspectives. Despite multiple nominations, the couple never received the Nobel Prize in Physiology or Medicine. Drawing upon archival sources from the Nobel Forum and the Vogt Archive in Düsseldorf, we reconstruct the history of their candidacies, the reasons why they were proposed, and those behind the committee’s repeated rejections. Their work on cyto- and myeloarchitectonics, the functional anatomy of the basal ganglia, and structure–function relationships in the cerebral cortex earned them international recognition. However, the Nobel Committee remained unconvinced, often citing issues of scientific priority, insufficient novelty, and the controversial nature of some of their claims. Despite their exclusion from the prize, the Vogts’ research shaped the development of brain science across Europe and beyond, influencing later Nobel laureates and contributing to foundational concepts in neuroanatomy and -physiology. Their case invites reflection on the historical contingencies of scientific recognition and the shifting criteria for what counts as a “discovery” worthy of the Nobel Prize.

## 1 Introduction

No other award worldwide has such a strong aura of scientific excellence as the Nobel Prize, first awarded in 1901. Thus, it is not surprising that commentators have analyzed various aspects around the prize (Liljestrand, 1962), ranging from the scientific and societal impact of the winners (Crawford, 2002), via the prize population (nationalities, minorities, the gender gap etc.), to the nomination campaigns of Nobel laureates (Hansson et al., 2019). In addition, the limited number of awards per year inevitably triggers discussions about the soundness of the choice, as well as, more or less justified, polemics about the exclusions (Bliss, 2007; Hansson, 2023).

Among those who were often mentioned as candidates for the prize were Oskar (1870–1959) and Cécile Vogt (1875–1962). They were repeatedly nominated for work on myeloarchitecture of the human cerebral cortex (Vogt and Vogt, 1919b) and the functional anatomy of the basal ganglia (e.g., Vogt and Vogt, 1919a; Satzinger, 1998). Cécile Vogt even as the first female nominee for the Nobel Prize in physiology or medicine prize category (Hansson and Fangerau, 2018). In addition, they acted as nominators themselves and had many international professional and social contacts in Nobel circles (laureates, nominees, nominators) – a network that has inspired recent scholarship on controversies regarding research ethics and politics (Calabrese and Selby, 2024).

A comprehensive analysis of their involvement in the Nobel circles is still lacking. Also lacking is an answer to the question why their nominators, despite of a powerful scientific network, did not succeed in convincing the Nobel Prize jury. Drawing on Nobel Prize nominations and reports by the Nobel Committee collected at the Nobel Forum in Solna, Sweden, and correspondences in the Cécile and Oskar Vogt archive at the Heinrich-Heine-University in Düsseldorf, Germany, we shed light on how they were enacted in the nominations, why parts of the prize jury did not view them as prize-worthy^1^ and, from today’s perspective, discuss their scientific contribution.

## 2 Nobel Prize nominations for Cécile and Oskar Vogt

**Figure 1:**
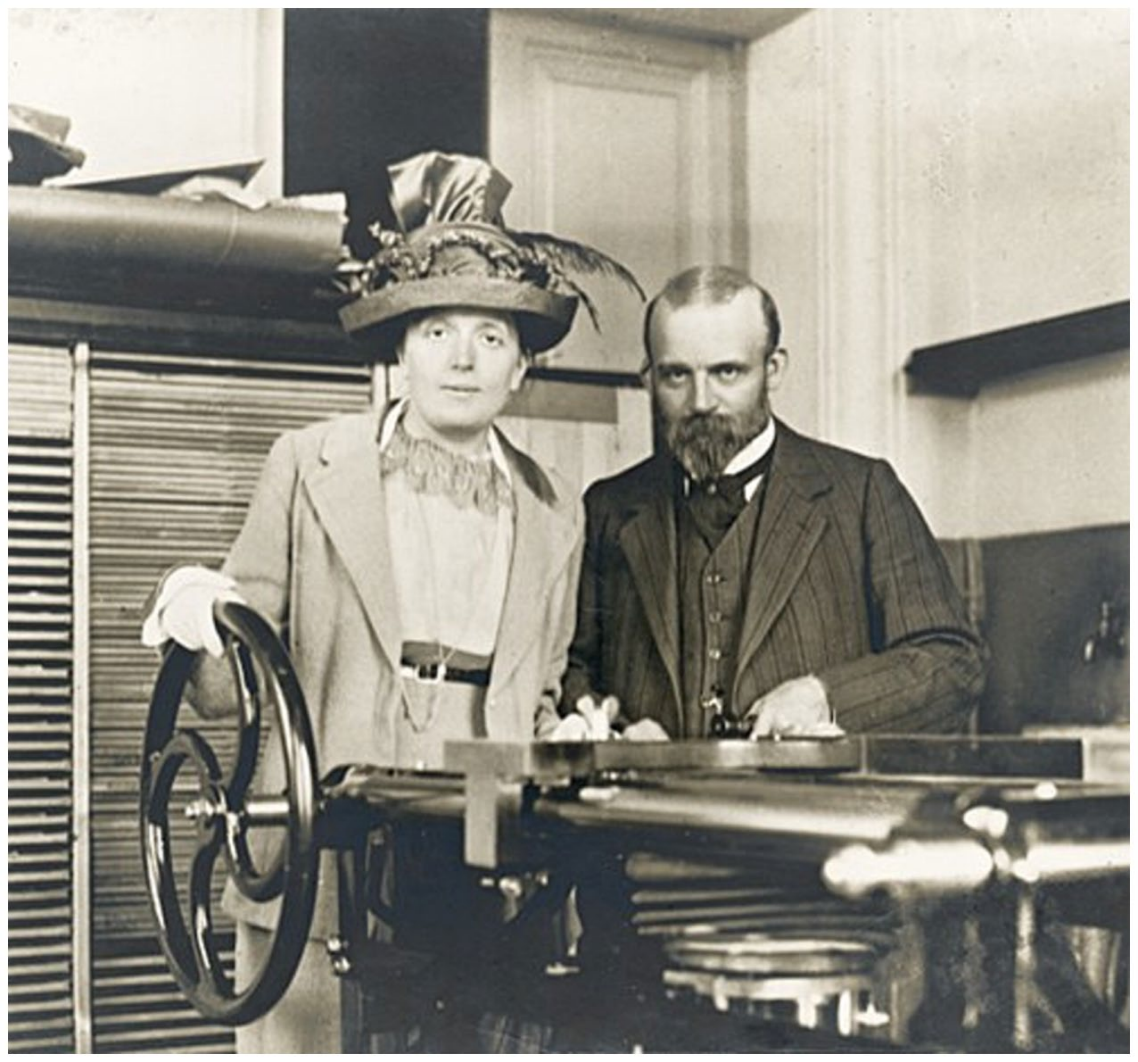
Cécile and Oskar Vogt with their Pantomikrotom, a microtome for large sections, in the institute (around 1905). Courtesy Vogt Archive, Düsseldorf.

Oskar and Cécile Vogt (née Mugnier) were prominent figures in many fields of science and medicine, including neurology, neurophysiology, ontogeny, neuroanatomy, psychiatry and hypnosis (Klatzo, 2002). They made significant contributions to brain research, e.g., myeloarchitectonics, cytoarchitectonics, and basal ganglia functional anatomy (Vogt, 1910; Vogt and Vogt, 1919a, 1954, 1956; see Figure 1). In 1898, they opened a psychiatric practice in Berlin, which included a privately funded Neurological Central Station. In 1902, this station became affiliated with Berlin University as a Neurobiological Laboratory, and later to grow into the *Kaiser Wilhelm Institute for Brain Research* in Berlin (1914), which Oskar headed until 1937. Following the inauguration of a new building in 1931, it was considered one of the largest and most modern of its kind in the world (Bielka, 1997; Martin et al., 2020; Marazia and Fangerau, 2018). The institute had departments of anatomy and electrophysiology, and included such diverse fields as experimental, population and human genetics, as well neurochemistry and pharmacology, towards a fully neuro-biological approach. At the same time, this research was linked to a clinical ward with up to 60 beds, to inform it by and align it with clinical observations, a concept that he early developed and that aimed to integrate basic research with clinical insights (Vogt, 1900, 1901). At the Vogt institute, generations of collaborators strived towards integrating physiological, anatomical and pathological observations on humans and animals in a genetic and evolutionary picture. Following Vogt’s forced retirement by the Nazi government in 1937, the Vogts continued their work at a private institute in Titisee, Neustadt, Black Forest (Kreutzberg et al., 1992).

Cécile and Oscar Vogt were proposed as Nobel Prize candidates between 1921 and 1956, most times as a duo. More than a dozen researchers from various countries nominated the couple, including two Nobel Prize laureates: Robert Bárány and António Egas Moniz.

The nomination (proposed by authoritative, invited personalities) is but the first step of a complicated process, followed by a comparative review of all the nominations (in the field of Physiology or Medicine, in this case), and the compilation of a shortlist of candidates for special examinations.

Cecile and Oskar Vogt were first nominated in 1921 (for the 1922 Prize) by Robert Bárány, Emil Holmgren and Gustaf Bergmark, for the overall results of their cyto- and myeloarchitectonic, as well as pathological work, summarised in “Allgemeinere Ergebnisse unserer Hirnforschung” (Vogt and Vogt, 1919b) and “Zur Lehre der Erkrankungen des striären Systems” (Vogt and Vogt, 1920). On this occasion, the Vogts made it to the shortlist, with the Italian neuropathologist Leonardo Bianchi, the Danish pathologist Johannes Fibiger, the American biochemist Edward Calvin Kendall, the US-Austrian hematologist Karl Landsteiner and the German physiologist Rudolf Magnus. Cécile and Oskar Vogt were evaluated by the Stockholm neurologist Frithiof Lennmalm with a so-called “special investigation,” a report that aimed at settling fundamental questions, such as the legitimacy of their work, issues of scientific priority, and the impact of their work on later research. In his report, Lennmalm concluded that Vogt’s publications were “extraordinarily meritorious” [utomordentligt förtjänstfulla] and that the scope of the results is significant, “but,” he added, “they lack scientific priority.” Thus, his verdict was negative: “Several researchers have previously found changes in the striatum and pallidum in various listed diseases. […] Under such circumstances, I do not think that the Nobel Prize should be awarded to the Vogts for their work on the striate system.”^2^ The last stage in the decision process is the (unprotocolled) discussion, in which the final decision is reached. The 1922 Nobel Prize in Physiology or Medicine was divided equally between Archibald Vivian Hill “for his discovery relating to the production of heat in the muscle” and Otto Fritz Meyerhof “for his discovery of the fixed relationship between the consumption of oxygen and the metabolism of lactic acid in the muscle.”

Although hard to assess, the issue of priority has always been of the greatest relevance for the adjudication of the prize. Lennmalm’s evaluation was therefore not the best premise. On the other hand, it was not necessarily a damnation. Within the committee, opinions are freely exchanged on discoveries and discoverers that are often difficult to compare directly. An authoritative and effective patron inside the Committee could still have turned the situation in favour of the Vogts. Understandably, no records are kept of these confidential debates but, sometimes, alternative sources, like correspondences or (auto-)biographies, provide critical clues. Cécile and Oskar Vogt corresponded with several professors at the Karolinska Institute, such as Salomon Eberhard Henschen, his son Folke, and Emil Holmgren (Lindberg, 2013), but in this case it is Folke Henschen’s autobiography, which sheds light on the event. Henschen jr had the greatest consideration for the Vogts: “[there] are not many people who have played such a big role in my life,” he wrote in the late 1950s (Henschen, 1957; Björkman, 2016).

Despite the strict confidentiality rule of the Academy, both Folke and his father regularly updated Cécile and Oskar Vogt on their Nobel Prize chances in their letters. The Henschen-Vogt correspondence shows that the chances for a Nobel Prize for the pair did not improve over the years. Both Vogts have also been invited to nominate researchers by the Nobel committee and proposed Thomas H. Morgan and Herman Joseph Muller. In their nomination of 1932, Oskar Vogt emphasized Morgan’s research on genetics, gene mutations, phenotypical divergencies, the demonstration of three coupling groups in the genes of Drosophila, and “crossing over,” and Muller’s lethal factors, the demonstration of a fourth coupling group in the genes of Drosophila, and the effect of temperature changes and X-rays on the mutation rate. Both would later receive the award (Morgan in 1933, Muller in 1946).

Henschen jr also provided information on the “intense, even violent” discussion within the committee in 1922. According to his account, the Professor of histology Emil Holmgren had painstakingly prepared an endorsement of the Vogts, but had fallen ill before the decisive meeting: “he came to the jury meeting on October 12 with a bandage around his neck and forehead” Henschen later recounted. Reportedly, Holmgren collapsed during his speech, turned gray in the face and had to leave. Ten days later, he died at the age of 56. The Vogts did not only lose an ally in this meeting. Holmgren’s forced retirement from the dispute left the stage free for a very powerful opponent, Johan E. (Jöns) Johansson. Apart from being the “too-dominating chairman of the Nobel Committee” (Henschen, 1957, p. 199), Johansson had been personally involved in designing the prize regulations, and the one who, in his first year of chairmanship (1918) had for the first time introduced the now traditional reference to “discovery” in the prize motivation (Pahlm and Uvelius, 2019; Norrby, 2022).

During the following years, nominations kept coming in to the jury. Edmund Forster, Director of the Greifswald University Neurological Clinic, emphasized in his 1928 Nobel Prize nomination:

“*The researchers succeeded in proving that the cerebral cortex is divided into many fields whose function, as they were also able to confirm by experiments, is different. Despite the fact that the researchers went too far in interpreting their findings (and thus believed they could deduce a particularly great intelligence from the structure of Lenin’s cerebral cortex, whereas in reality the documents are not in the least sufficient for this assumption), their work is admirable and of the greatest importance and the authors are worthy of the Committee’s attention.”*^3^

After Lenin’s death in 1924, Vogt (together with S. E. Henschen) was invited to study his brain (see Richter, 2000 for the complete story). Since the Soviet government would not allow Lenin’s brain to leave the USSR, the Vogts had to move repeatedly for longer periods to Moscow, where they founded a brand-new brain research centre on the blueprint of their Kaiser-Wilhelm-Institute on a lesser scale. In addition to their interdisciplinary approach, the Vogts contributed equipment (e.g., microtomes, optic banks) and protocols for histological processing and documentation (Vogt, 1929). Later, the new institute undertook brain mapping by the techniques of cyto-, myelo- and vascular architecture, complemented by research into the ontogeny and inter-subject variability of the brain at the microstructural level (Sarkisov et al., 1949). If the involvement with Lenin’s brain had given unprecedented visibility to the Vogts, their techniques, and their accomplishments, and strengthened their relationship with Soviet science (especially genetics, see Satzinger, 1998; Calabrese and Shamoun, 2025), their interpretation of the results had attracted many critiques in academic circles (Hagner, 2004).

Notably, Oskar Vogt delivered a speech on 11 November 1929 at the Moscow Institute (Vogt, 1929), which was published in the same year (Vogt, 1929). Therefore, the paper is not purely a research paper, and it does not only concern Lenin’s brain, but also introduces the methods that have been applied, other ideas such as so-called “elite-brains” and also includes more political statements (e.g., thanking collaborators and supporters). Vogt was interested in the physiological interpretation of cytoarchitecture and believed that researching so-called elite brains was a way to address this question. He placed his research on Lenin’s brain in the context of research on other so-called elite brains. Regarding Lenin’s brain, Vogt reported a high number of particularly large pyramidal cells in cortical layer III. These cells were seen as “association cells” that connect one area to other brain areas. His conclusion that Lenin was an “association athlete” (page 110, Vogt, 1929) seems strange from today’s perspective and is not sufficiently supported by experimental findings. However, one could argue that Vogt was in an extremely difficult situation, having to balance scientific insights with political caution at a time when Stalin became increasingly powerful after he had eliminated his opponents. One could argue that this one-sided focus on Lenin’s brain does not do justice to Vogt’s scientific achievements.

To mention it in a letter of nomination was arguably not a smart move by the nominator. The committee members discussed the Vogt’s work on the segregation and structure of the cerebral cortex and the breadth of their research interests, ranging from hypnosis to the anatomical dissection of “famous” brains (Hagner, 2003).

The study of so-called “elite brains” had accompanied the growth of brain research from the second half of the 19^th^ century, as a widely accepted approach to the understanding of structure–function relations, and a “physiological” counterpart to the study of pathological lesions (Hagner, 2004). Vogt aimed to understand the so-called normo-anatomy plus function in animals and humans before studying pathologies, so-called abnormal human brains or brains of outstanding persons (Vogt and Vogt, 1956). This may explain why he did not follow up with systematic studies on “famous” brains.

**Figure 2:**
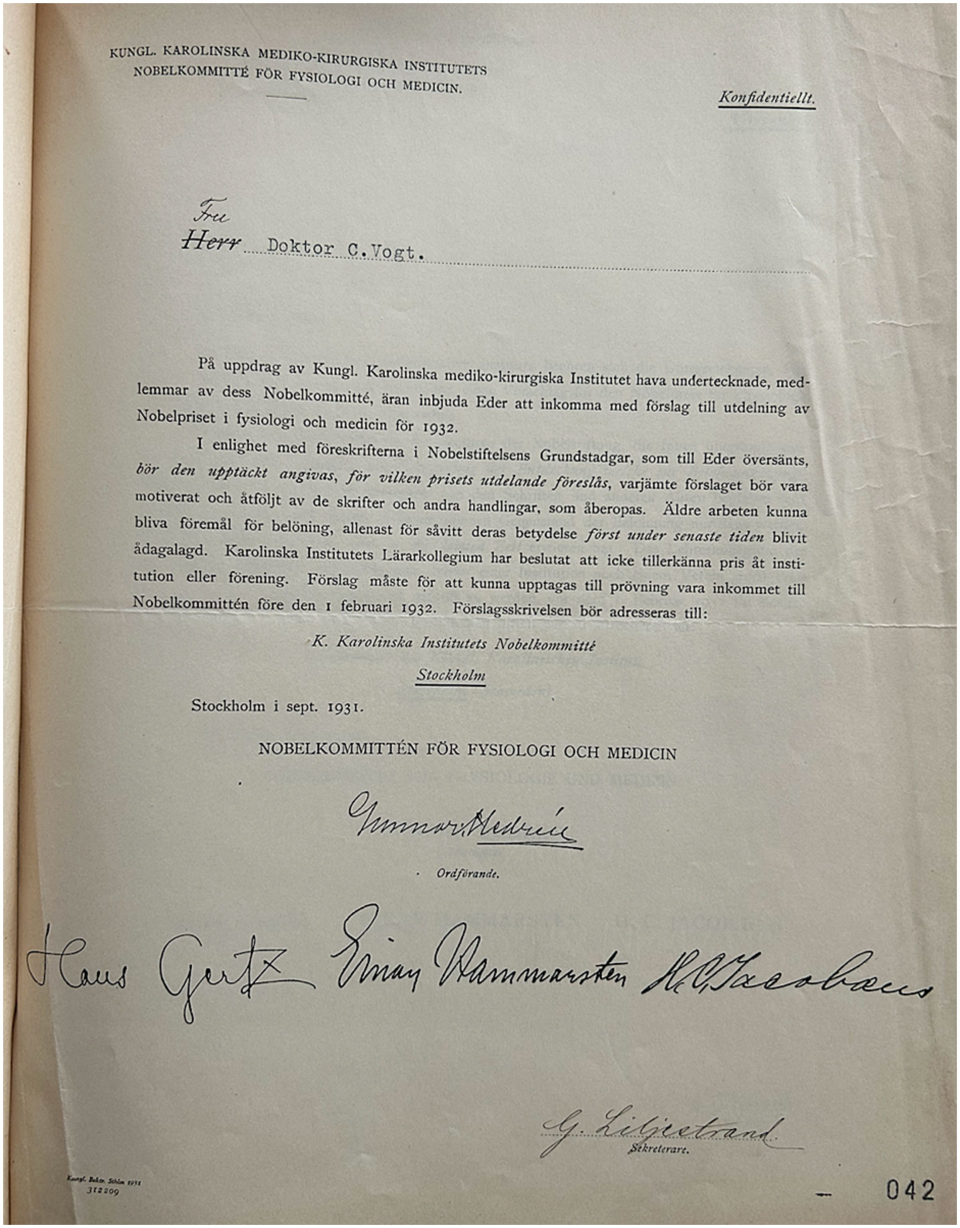
Personal nomination invitation to Vogt (1932) is archival material from the Vogt archive. Courtesy of the Cécile and Oskar Vogt Archive, Düsseldorf. Correspondence with the Nobel foundation 1932.

In 1937, an individual nomination for Oskar came by the German Otfrid Foerster. A neurologist and neurosurgeon, Foerster had an approach converging with that of the Vogts: operating under local anesthesia, he was able to study sensorimotor functions in awake patients by means of electric stimulation.^4^ Vogt was very much interested in Forester’s observations, which promised to provide a physiological test of the myeloarchitectonic parcellation (Vogt and Vogt, 1926; *Cf.* Figure 2). This was supplemented by a comparative approach in the Vogt lab, where stimulation was used to study sensorimotor function and subsequently the underlying brain architecture.

This time, the evaluation fell on the Stockholm neurologist Nils Antoni, who had visited the Vogt Institute in Berlin. He wrote that, since Müller’s report of the Vogts in 1922, O. Vogt had not produced much new, with the possible exception of the studies of eye movement fields, but that some of their older research had been confirmed more recently by scholars such as the nominator Foerster himself (Palmen et al., 2022). Antoni’s succinct report ended on a skeptical note: “I am unsure whether Vogt’s results have the degree of novelty, consistent certainty or scope, that an admission to a special investigation can be considered justified,” meaning that the Vogt’s did not reach the Nobel shortlist in 1937.^5^

**Figure 3:**
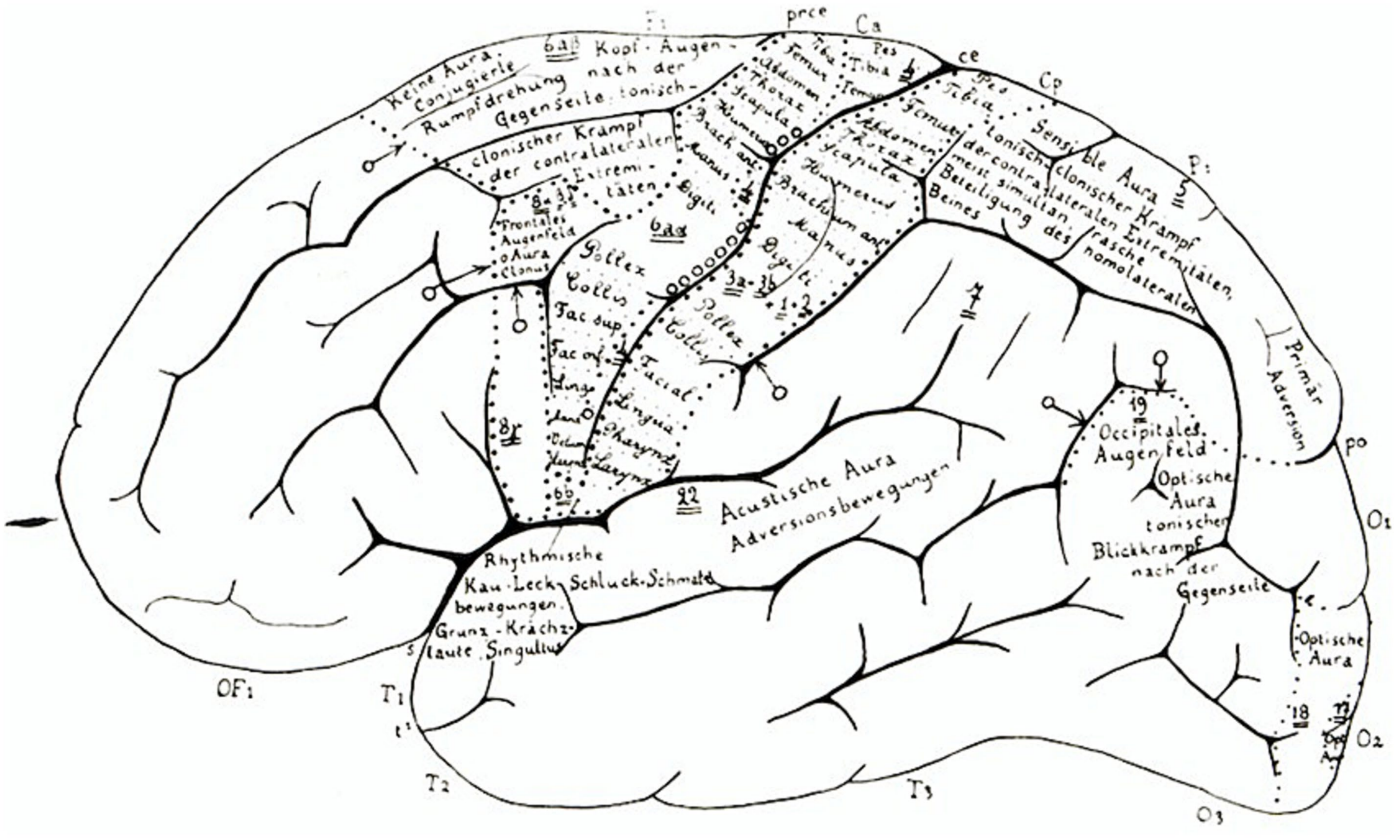
Reproduced from Vogt and Vogt (1926). Courtesy of the Cécile and Oskar Vogt Archive, Düsseldorf. Left hemisphere lateral surface view, with selected annotations.

In the following years the friends and supporters of the Vogt’s did not become tired and kept on nominating them. The most persistent of all Vogt nominators (in 1923, 1931, 1950, 1956) was the Frankfurt neurologist and psychiatrist Karl Kleist, who himself – inspired by them – was interested in the architectonics of the brain. His admiration was no secret in the scientific community, since Kleist also published several hagiographic accounts about his role models in scientific journals (e.g., Kleist, 1950) (Figure 3).

Kleist’s last nomination of both Vogt and Vogt (1956), and the 1952 nomination of Oskar alone by the Portuguese neurologist António Caetano de Abreu Freire Egas Moniz (Nobel Prize in Physiology or Medicine 1949) deserve some special attention, and can be read in parallel.^6^ They are both exceptionally long, and both try to do justice not only to the experimental contributions of the nominees, but also to the impact they had on the scientific and medical field. Both nominations highlight the imposing continuity and logical rigor of the experimental design of the Vogts: their myelo- and cytoarchitectonic studies (together with their collaborator Korbinian Brodmann, cf. Brodmann, 1909), substantiated by electrophysiological experiments; the evolutionary significance of their comparative approach; their consistent attention to the genetics of variation and mutation, as a pendant to anatomy and physiology. Finally, both nominators strongly underscore the medical implications of their research, especially of the introduction of the concept of pathoclisis (selective vulnerability) and its application to a number of diseases, most notably schizophrenia. Despite important differences of emphasis (Kleist stressing the pathological implications, Moniz the psychological, and even philosophical ones), both nominators successfully tried to convey the ambitiousness of Cecile and Oskar Vogt’s vision. Just as important as the combination of different approaches and techniques (anatomical, physiological and pathological, cf. Vogt and Vogt, 1902), was to them the attention to the downstream applicability of their basic science, in medical psychology, eugenics, and psychiatry (see, e.g., Vogt, 1911).

Kleist argued in his 1956 nomination:

“*[.] I recommend that Mr. Oskar Vogt and Mrs. Cécile Vogt be awarded the Prize for Physiology and Medicine for their achievements in elucidating the pathoanatomical basis of schizophrenia and consider this to be the most important discovery of recent years in the field of physiology and medicine […] the prize could also be awarded in the sense of paragraph 2 of the statutes for the entire scientific life’s work of the research couple, in which discoveries made some time ago only became significant later and even more recently. With the highest esteem Prof. Dr. K. Kleist*”^7^.

Although parts of the Nobel Prize nominations for the Vogts were full of admiration for their multifaceted brain research, the nominators could not convince the committee members that their discoveries merited a prize, a fate they share with other internationally renowned neurologists and psychiatrists like Emil Kraepelin, Henry Head and Vladimir Bektherev (Hansson et al., 2020). Oskar Vogt died in 1959, Cécile Vogt 3 years later in Cambridge. Although they never received the Nobel Prize, the international impact of the Vogt-Vogt-school (Nieuwenhuys and Broere, 2023) led to many other accolades, honorary doctorates and awards.

## 3 Contemporary developments

Regarding the architectonic approach that was in the center of Vogts’ ideas about localization of function – is this research still present and useful? Korbinian Brodmann, an employee and colleague of the Vogts created a cytoarchitectonic map (Brodmann, 1909), which is still present in today’s literature and part of various atlases and digital repositories of the human brain (for an overview see Amunts and Zachlod, 2025, Zilles and Amunts, 2010). Cytoarchitectonic maps serve as microstructural reference in modern multi-modal atlas at EBRAINS.^8^ However, the field has changed significantly since Brodmann and the Vogts mapped the cerebral cortex (Zilles and Amunts, 2010; Amunts and Zilles, 2015). Instead of 43 cortical areas, independent research suggests that the cerebral cortex can be subdivided into about 200 areas (Glasser et al., 2016; Amunts et al., 2020). More importantly, some of Brodmann’s areas do not represent functionally meaningful units (e.g., Brodmann’s area 19 of visual cortex), and intersubject variability is seen as a central factor of any mapping approach (Zilles and Amunts, 2010; Amunts and Zilles, 2015).

Similar arguments hold true when comparing Vogts’ myeloarchitectonic map with modern findings. The interest in the myeloarchitecture increased with the advance of MR imaging due to the correlation of the MR-signal to the underlying myeloarchitecture (see for instance Eickhoff et al., 2005; Nieuwenhuys and Glasser, 2024; Turner, 2019; Walters et al., 2007). Notably, research in 20 published papers by the Vogts was re-analyzed by Rudolf Nieuwenhuys, who acknowledged the relevance of the historical map for modern neuroimaging. In order to compare the maps of Vogts with neuroimaging data, the two-dimensional drawings were projected into a three-dimensional space (Nieuwenhuys and Broere, 2023). Just recently, a comparison of the multimodal MRI-based parcellation of Glasser and van Essen (2011) and the myeloarchitectonic parcellation of the Vogt’s as presented by Nieuwenhuys and Broere (2023) has been published to better understand the concordances between these two maps (Nieuwenhuys and Glasser, 2024). Nieuwenhuys and Broere referred to the myeloarchitectonic map with 182 areas, which is, when looking to the total number of areas, rather close to that of, for example, Glasser and Van Essen (2011) or Amunts et al. (2020). However, when going into the details, discrepancies are evident. While the Vogts have identified, for example, 6 areas in the insula, recent methods show at least twice as much in the multimodal brain atlas at EBRAINS.^9^ At the same time, many of the 63 temporal areas of the Vogt-map have not been confirmed by more recent research. With 3D Polarized Light Imaging, an optical technique that visualizes axons and thin fiber bundles, a method is available that brings Vogt’s vision about myeloarchitecture into the three-dimensional space (Axer and Amunts, 2022), although a complete map still remains a project of future research.

The Vogts’ histological collection is still available in Düsseldorf and is being digitized to make their legacy accessible to a wider research community; for more information about the collection, a list of scientific papers around the collection and to get in contact.^10^ In their professional life, the Vogts have collected and processed over sixty years approximately 850.000 sections of human brains and brains of more than 100 non-human primates and other species. These sections are accompanied by documents, protocols, photographs, clinical records and scientific papers and other objects. The digitalization of this collection would allow to open the collection to a broader science community, go back to the original histological data and unstained material and to prove their validity by more recent methods. It would also allow to investigate the historical, sociological, ethical and political aspects of 20th-century science. Eventually, it will open up new deeper insights into the validity of Oskar and Cécile’s research ideas, to better understand whether their research was “extraordinarily meritorious,” but “lack scientific priority” (Lennmalm, 1922). One characteristic of good research is that it stands the test of time and inspires other research. From the authors’ point of view, this is certainly true of the Vogt Collection, regardless of whether it wins the Nobel Prize.

This year, we are celebrating the 150th and 155th birthdays, respectively of the two pioneers of modern brain research, who made important contributions at the level of concepts and methods, with major impact in the field. The decisions of the Nobel Committee are part of the history of science. What we can do today is try to use the scientific legacy of the Vogts and build on it.

## Footnotes

^1^The Nobel archive keeps the nomination dossiers and committee evaluations of all proposed scholars. Only invited scholars by the Nobel Committee can submit eligible nomination letters, but professors at medical faculties in Scandinavia and previous Nobel laureates (physiology or medicine) may propose candidates each year. According to the Nobel statutes, there is an embargo of 50 years before historians can view the files.

^2^Nobel archive, Lennmalm (yearbook 1922).

^3^Nobel archive, Forster (yearbook 1928). Unless otherwise stated, all translations are by the authors.

^4^Foerster approach was later developed by his Canadian colleague Wider Penfield, who became famous for the “homunculus” showing the somatotopy of motor and somatosensory control (*Cf.* Penfield and Boldrey, 1937).

^5^Nobel archive, Antoni (yearbook 1937). The comparison of electro-physiological findings with the microscopical anatomy, i.e., myeloarchitecture, can be seen as one of the first multi-modal studies to study structure–function relationship in the human brain– an approach that was later extended with neuroimaging techniques not only in patients but also in healthy volunteers. In that respect, Vogt’s early comparison can be seen as pioneering.

^6^Nobel archive, Moniz (yearbook 1952); Nobel archive, Kleist (yearbook 1956).

^7^Nobel archive, Kleist (yearbook 1956).

^8^ https://www.ebrains.eu/tools/human-brain-atlas

^9^ https://search.kg.ebrains.eu/?category=Dataset&q=insula

^10^ https://www.uniklinik-duesseldorf.de/patienten-besucher/klinikeninstitutezentren/c-u-o-vogt-institut-fuer-hirnforschung/sammlungen

## References

[ref1] Amunts K. Mohlberg H. Bludau S. Zilles K. (2020). Julich-brain: a 3d probabilistic atlas of the human brain’s cytoarchitecture. Science 369, 988–992. doi: 10.1126/science.abb4588, PMID: 32732281

[ref2] Amunts K. Zachlod D. (2025). “Human brain anatomy and atlases” in Encyclopedia of the human brain. ed. Grafman J. H. (Amsterdam: Elsevier), 325–345.

[ref3] Amunts K. Zilles K. (2015). Architectonic mapping of the human brain beyond Brodmann. Neuron 88, 1086–1107. doi: 10.1016/j.neuron.2015.12.001, PMID: 26687219

[ref4] Axer M. Amunts K. (2022). Scale matters: the nested human connectome. Science 378, 500–504. doi: 10.1126/science.abq2599, PMID: 36378967

[ref5] Bielka H. (1997). “Kaiser-Wilhelm-Institut für Hirnforschung 1930–1945” in Die Medizinisch-Biologischen Institute Berlin-Buch: Beiträge zur Geschichte. ed. Bielka H. (Berlin, Heidelberg: Springer Berlin Heidelberg), 18–39.

[ref6] Björkman M. (2016). “Ras, vetenskap och objektivitet.: Några lojaliteter hos Folke Henschen” in De intellektuellas förräderi?: Utbyte mellan Sverige och Tyskland under Tredje Riket. eds. Björkman M. Lundell P. Widmalm S. (Lund: Arkiv förlag & tidskrift), 161–182.

[ref7] Bliss M. (2007). The discovery of insulin. 25th Anniversary Edn. Chicago, IL: Chicago University Press.

[ref8] Brodmann K. (1909). Vergleichende Lokalisationslehre der Grosshirnrinde in ihren Prinzipien dargestellt auf Grund des Zellenbaues. Leipzig: Verlag von Johann Ambrosius Barth.

[ref9] Calabrese E. J. Selby P. B. (2024). Newly discovered letter: why Muller failed to cite the negative mouse mutation findings of Snell, preserving his chances to receive the Nobel prize. Arch. Toxicol. 98, 2739–2741. doi: 10.1007/s00204-024-03807-1, PMID: 38909170

[ref10] Calabrese E. J. Shamoun D. Y. (2025). The unraveling of a Nobel prize: how Hermann Muller was awarded the Nobel prize: a front for eugenics. J. Occup. Environ. Hyg. 22, 149–168. doi: 10.1080/15459624.2024.2440558, PMID: 39761211

[ref12] Crawford E. (Ed.) (2002). Historical studies in the Nobel archives: The prizes in science and medicine. Tokyo: Universal Academy Press.

[ref13] Eickhoff S. Walters N. B. Schleicher A. Kril J. Egan G. F. Zilles K.. (2005). High-resolution MRI reflects myeloarchitecture and cytoarchitecture of human cerebral cortex. Hum. Brain Mapp. 24, 206–215. doi: 10.1002/hbm.20082, PMID: 15543596 PMC6871702

[ref14] Glasser M. F. Coalson T. S. Robinson E. C. Hacker C. D. Harwell J. Yacoub E.. (2016). A multi-modal parcellation of human cerebral cortex. Nature 536, 171–178. doi: 10.1038/nature18933, PMID: 27437579 PMC4990127

[ref15] Glasser M. F. Van Essen D. C. (2011). Mapping human cortical areas in vivo based on myelin content as revealed by T1- and T2-weighted MRI. J. Neurosci. 31, 11597–11616. doi: 10.1523/JNEUROSCI.2180-11.2011 21832190 PMC3167149

[ref16] Hagner M. (2003). “Im Pantheon der Gehirne. Die Elitegehirnforschung von Oskar und Cécile Vogt” in Rassenforschung an Kaiser-Wilhelm-Instituten vor und nach 1933. ed. Schmuhl H. W. (Göttingen: Wallstein), 99–144.

[ref17] Hagner M. (2004). Geniale Gehirne: Zur Geschichte der Elitegehirnforschung. Göttingen: Wallstein.

[ref18] Hansson N. (2023). Wie man keinen Nobelpreis gewinnt - Die verkannten Genies der Medizingeschichte. München: Gräfe und Unzer.

[ref19] Hansson N. Fangerau H. (2018). Female physicians nominated for the Nobel prize 1901–50. Lancet 391, 1157–1158. doi: 10.1016/S0140-6736(18)30576-2, PMID: 29525409

[ref20] Hansson N. Halling T. Fangerau H. (2019). “Introduction” in Attributing excellence in medicine: The history of the Nobel prize. eds. Hansson N. Halling T. Fangerau H. (Leiden: Brill), 1–14.

[ref21] Hansson N. Palmen L. Padrini G. Karenberg A. (2020). Babinski, Bektherev, Cerletti, head, and Hitzig: European neurologists nominated for the Nobel prize 1901-1950. Eur. Neurol. 83, 542–549. doi: 10.1159/000509078, PMID: 32731244

[ref22] Henschen F. (1957). Min långa väg till Salamanca. En läkares liv. Stockholm: Bonniers Förlag.

[ref23] Klatzo I. (2002). Cécile and Oskar Vogt. The visionaries of modern neuroscience. Cham: Springer Science & Business Media. 12187846

[ref24] Kleist K. (1950). Oskar Vogt 80 Jahre, Cécile Vogt 75 Jahre. Arch. F. Psychiatr. U. Z. Neur. 185, 619–623. 10.1007/BF00935512 14800361

[ref25] Kreutzberg G. W. Klatzo I. Kleihues P. (1992). Oskar and Cécile Vogt, Lenin's brain and the bumble-bees of the Black Forest. Brain Pathol. 2, 363–371. doi: 10.1111/j.1750-3639.1992.tb00712.x, PMID: 1341969

[ref26] Lennmalm F. (1922). Report on behalf of the Nobel Committee. Solna: Archive of the Nobel Committee for physiology or medicine.

[ref27] Liljestrand G. (1962). “The prize in physiology or medicine” in Nobel: The man and his prizes. eds. Schück H. Sohlman R. Österling A. Liljestrand G. Westgren A. Siegbahn M. (Amsterdam: Elsevier Publishing Co.).

[ref28] Lindberg B. S. (2013). Salomon Eberhard Henschen: en biografi. Uppsala: Acta Universitatis Upsaliensis.

[ref29] Marazia C. Fangerau H. (2018). Imagining the brain as a book: Oskar and Cécile Vogt's “library of brains”, vol. 243: Progress in Brain Research, 181–203. 10.1016/bs.pbr.2018.10.012 30514523

[ref30] Martin M. Karenberg A. Fangerau H. (2020). Neurowissenschaftler am Kaiser-Wilhelm-Institut für Hirnforschung im Dritten Reich: Oskar Vogt – Hugo Spatz – Wilhelm Tönnis. Nervenarzt 91, 89–99. doi: 10.1007/s00115-019-00847-2 32067090

[ref31] Nieuwenhuys R. Broere C. A. J. (2023). A new 3D myeloarchitectonic map of the human neocortex based on data from the Vogt-Vogt school. Brain Struct. Funct. 228, 1549–1559. doi: 10.1007/s00429-023-02671-6, PMID: 37378856 PMC10751253

[ref32] Nieuwenhuys R. Glasser M. F. (2024). A comparison of two maps of the human neocortex: the multimodal MRI-based parcellation of Glasser et al.(2016a), and the myeloarchitectonic parcellation of Nieuwenhuys and Broere (2023), as a first step toward a unified, canonical map. Brain Struct. Funct. 229, 2509–2521. doi: 10.1007/s00429-024-02860-x, PMID: 39576342 PMC11611935

[ref33] Norrby E. (2022). Nobel prizes: Genes, viruses and cellular signaling. Singapore: World Scientific.

[ref34] Pahlm O. Uvelius B. (2019). The winner takes it all: Willem Einthoven, Thomas Lewis, and the Nobel prize 1924 for the discovery of the electrocardiogram. J. Electrocardiol. 57, 122–127. doi: 10.1016/j.jelectrocard.2019.09.012, PMID: 31629994

[ref35] Palmen L. Eisenberg U. Karenberg A. Fangerau H. Hansson N. (2022). Ein zu internationaler Berühmtheit gelangter Forscher und Arzt: Otfrid Foerster (1873–1941) als Nobelpreiskandidat. Nervenarzt 93, 720–727. doi: 10.1007/s00115-021-01184-z 34524517 PMC9276572

[ref36] Penfield W. Boldrey E. (1937). Somatic motor and sensory representation in cerebral cortex of man as studied by electrical stimulation. Brain 60, 389–443.

[ref37] Richter J. (2000). Rasse, Elite, Pathos: eine Chronik zur medizinischen Biographie Lenins und zur Geschichte der Elitegehirnforschung in Dokumenten. Neuere Medizin- und Wissenschaftsgeschichte. Quellen und Studien, Vol. 8. Herboisheim: Centaurus Verlag.

[ref38] Sarkisov S. A. Filimonoff I. N. Preobrashenskaya N. S. (1949). Cytoarchitecture of the human cortex Cerebri. Moscow: Medgiz.

[ref39] Satzinger H. (1998). Die Geschichte der genetisch orientierten Hirnforschung von Cécile und Oskar Vogt in der Zeit von 1895 bis ca. 1927. Stuttgart: Deutscher Apotheker-Verlag.

[ref40] Turner R. (2019). Myelin and modeling: bootstrapping cortical microcircuits. Front. Neural Circuits 13:34. doi: 10.3389/fncir.2019.00034, PMID: 31133821 PMC6517540

[ref41] Vogt O. (1900). Über die Errichtung neurologischer Zentralstationen. Z. Hypn. 10, 170–177.

[ref42] Vogt O. (1901). Ueber centralisiertes hirnanatomisches Arbeiten. Bergmann: Verhandlungen des Kongresses für Innere Medizin. Wiesbaden, 498–502.

[ref43] Vogt O. (1910). Die myeloarchitektonische Felderung des menschlichen Stirnhirns. J. Psychol. Neurol. (Lpz.) 15, 221–232.

[ref44] Vogt O. (1911). La nouvelle division myeloarchitecturale de l’ecorce cerebrale et ses rapports avec la physiologie et la psychologie. J. Psychol. Neurol. 17, 1–15.

[ref45] Vogt O. (1929). Erster Bericht über die Arbeiten des Moskauer Staatsinstituts für Hirnforschung. J. Psychol. Neurol. (Lpz.) 40, 108–118.

[ref46] Vogt C. Vogt O. (1902). Zur Erforschung der Hirnfaserung. Denkschriften der Medicinisch-Naturwissenschaftlichen Gesellschaft. IX Band: Oskar Vogt, Neurobiologischen Arbeiten. Erste Serie: Beiträge zur Hirnfaserlehre. Jena: Verlag von Gustav Fischer, 3–145.

[ref47] Vogt C. Vogt O. (1919a). Erster Versuch einer pathologisch-anatomischen Einteilung striärer Motilitätsstörungen nebst Bemerkungen über seine allgemeine wissenschaftliche Bedeutung. J. Psychol. Neurol. (Lpz.) 24, 1–19.

[ref48] Vogt C. Vogt O. (1919b). Allgemeinere Ergebnisse unserer Hirnforschung; dritte Mitteilung: die architektonische Rindenfelderung im Lichte unserer neuesten Forschungen. J. Psychol. Neurol. (Lpz.) 25, 361–376.

[ref49] Vogt C. Vogt O. (1920). Zur Lehre der Erkrankungen des striären Systems. J. Psychol. Neurol. (Lpz.) 25, 627–846.

[ref50] Vogt C. Vogt O. (1926). Die vergleichend-architektonische und die vergleichend-reizphysiologische Felderung der Großhirnrinde unter besonderer Berücksichtigung der menschlichen. Naturwissenschaften 14, 1192–1195.

[ref51] Vogt C. Vogt O. (1954). Gestaltung der topistischen Hirnforschung und ihre Förderung durch den Hirnbau und seine Anomalien. J. Hirnforsch. 1, 1–46. doi: 10.1515/9783112526828-001

[ref52] Vogt C. Vogt O. (1956). Weitere Ausführungen zum Arbeitsprogramm des Hirnforschungsinstitutes in Neustadt/Schwartzwald. J. Hirnforsch. 2, 403–427. 13385469

[ref53] Walters N. B. Eickhoff S. B. Schleicher A. Zilles K. Amunts K. Egan G. F.. (2007). Observer-independent analysis of high-resolution MR images of the human cerebral cortex: in vivo delineation of cortical areas. Hum. Brain Mapp. 28, 1–8. doi: 10.1002/hbm.20267, PMID: 16773636 PMC6871284

[ref54] Zilles K. Amunts K. (2010). Centenary of Brodmann's map - conception and fate. Nat. Rev. Neurosci. 11, 139–145. doi: 10.1038/nrn2776 20046193

